# The guanine nucleotide exchange factor Arhgef7/βPix promotes axon formation upstream of TC10

**DOI:** 10.1038/s41598-018-27081-1

**Published:** 2018-06-11

**Authors:** Alejandro López Tobón, Megalakshmi Suresh, Jing Jin, Alessandro Vitriolo, Thorben Pietralla, Kerry Tedford, Michael Bossenz, Kristina Mahnken, Friedemann Kiefer, Giuseppe Testa, Klaus-Dieter Fischer, Andreas W. Püschel

**Affiliations:** 1 Institut für Molekulare Zellbiologie, Westfälische Wilhelms-Universität, Schloßplatz 5, D-48149, Münster, Germany; 2 Cells-in-Motion Cluster of Excellence, University of Münster, D-48149 Münster, Germany; 3 Department of Oncology and Hemato-Oncology, University of Milan, Milan, 20122 Italy; 4 European Institute of Oncology, Via Adamello 16, 20139 Milan, Italy; 5 Institut für Biochemie und Zellbiologie, Otto-von-Guericke-University, Medical Faculty, Leipziger Str. 44, 39120, Magdeburg, 39120 Germany; 6 Max-Planck-Institute for Molecular Biomedicine, Mammalian cell signaling laboratory, Röntgenstr. 20, D-48149 Münster, Germany; 7 European Institute for Molecular Imaging, Westfälische Wilhelms-Universität, Waldeyerstr. 15, D-48149 Münster, Germany

## Abstract

The characteristic six layers of the mammalian neocortex develop sequentially as neurons are generated by neural progenitors and subsequently migrate past older neurons to their final position in the cortical plate. One of the earliest steps of neuronal differentiation is the formation of an axon. Small GTPases play essential roles during this process by regulating cytoskeletal dynamics and intracellular trafficking. While the function of GTPases has been studied extensively in cultured neurons and *in vivo* much less is known about their upstream regulators. Here we show that Arhgef7 (also called βPix or Cool1) is essential for axon formation during cortical development. The loss of Arhgef7 results in an extensive loss of axons in cultured neurons and in the developing cortex. Arhgef7 is a guanine-nucleotide exchange factor (GEF) for Cdc42, a GTPase that has a central role in directing the formation of axons during brain development. However, active Cdc42 was not able to rescue the knockdown of Arhgef7. We show that Arhgef7 interacts with the GTPase TC10 that is closely related to Cdc42. Expression of active TC10 can restore the ability to extend axons in Arhgef7-deficient neurons. Our results identify an essential role of Arhgef7 during neuronal development that promotes axon formation upstream of TC10.

## Introduction

The characteristic six-layered structure of the mammalian neocortex arises by the sequential generation of neurons from neural progenitors located in the ventricular (VZ) and subventricular zone (SVZ) of the embryonic cortex and their subsequent radial migration into the cortical plate^1^. When they move from the VZ/SVZ into the intermediate zone, the newborn neurons initially have a multipolar morphology with several dynamic neurites^1^. After forming an axon and a leading process, neurons become bipolar and migrate into the cortical plate. A similar process can be observed in cultures of neurons from the embryonic brain^2^. Initially unpolarized neurons attach to the culture substrate (stage 1) and extend several neurites (stage 2). Neurons polarize by selecting one of these undifferentiated neurites as the axon (stage 3), which undergoes a rapid extension and acquires axon-specific markers.

Small GTPases play a crucial role during the transition from a multipolar to a bipolar morphology and the formation of axons^1,3,4^. Their activity is regulated by GEFs and GTPase activating proteins. The Rap1 GTPases are central regulators of the multi-to-bipolar transition^5^. They act upstream of Rho family GTPases like Cdc42 that is essential for the establishment of neuronal polarity and axon formation^6,7^. A knockout of Cdc42 results in an almost complete loss of axons in the cortex. Cdc42 directly regulates actin dynamics through cofilin^6^. The Par3/Par6 complex in addition couples Cdc42 to the GEFs Tiam1 and Tiam2/Stef that promote axon growth by activating Rac^2^. Despite its central role for neuronal development and axon formation, very little is known about the GEFs that regulate Cdc42 during neuronal differentiation.

**Figure 1:**
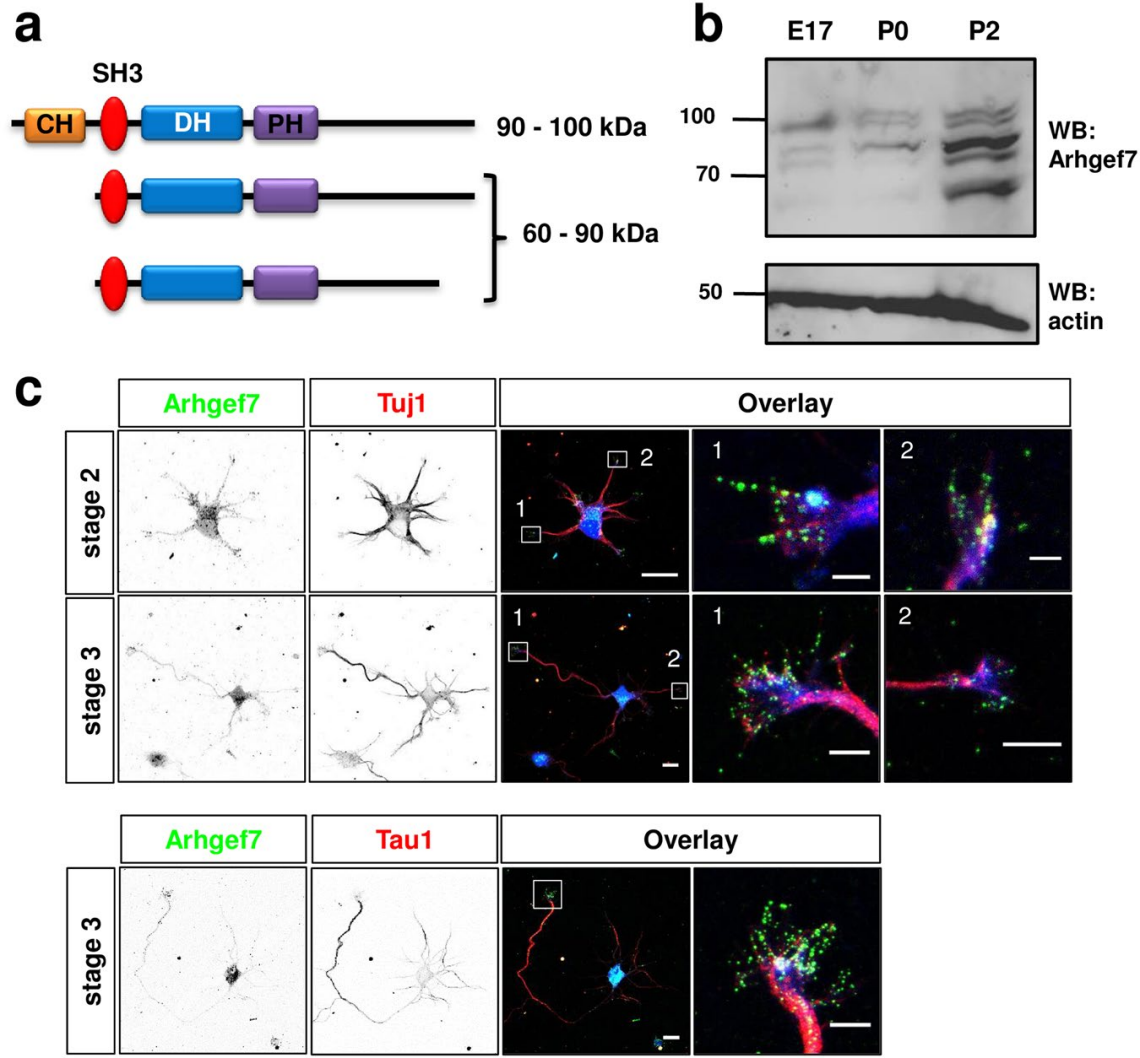
Expression of Arhgef7 in neurons. (**a**) A schematic representation of the Arhgef7 domain structure and the major isoforms is shown. DH: DBL homology domain, PH: plekstrin homology domain, CH: calponin homology domain, SH3: Src homology 3 domain, GBD: Git binding domain, CC: Coiled coil domain. (**b**) The expression of Arhgef7 in the cortex of E17 mouse embryos and postnatal mice (P0 or P2) was analyzed by Western blot (WB) using an anti-Arhgef7 antibody. The loading of comparable amounts of proteins was verified using and anti-actin antibody. Molecular weights are indicated in kDa. (**c**) Hippocampal neurons from E18 rat embryos were analyzed at 24 h (stage 2) or 72 h (stage 3) of culture by staining with an anti-Arhgef7 (green), the Tuji 1 or Tau-1 (red) antibodies and CellTracker Blue CMAC (blue) as a volume marker. The scale bars are 20 μm and 2 (upper panel) or 5 μm (lower panels), respectively.

The Rho guanine nucleotide exchange factor 7 (Arhgef7) also called βPix (Pax-interacting exchange factor beta) or Cool1 (Cloned out of library 1) and the closely related Arhgef6 (αPix) belong to the Dbl family of Rho GEFs. They contain a DH-PH (DBL and plekstrin homology) domain and activate Rac1 and Cdc42^8–14^. While a knockout of Arhgef6 is viable and shows defects in the immune system, the knockout of Arhgef7 is embryonically lethal at early stages of development precluding an analysis of its function in the nervous system^15,16^. In addition to the DH-PH domain that is responsible for its GEF activity, Arhgef7 also contains a CH (calponin homology) and an SH3 (Src homology 3) domain at the N-terminus and proline-rich, GIT1-binding and coiled coil (CC) domains at the C-terminus (Fig. 1a)^11,14,17,18^. These domains mediate the interaction with multiple binding partners that include p21 activated kinases (Paks), Git1 (G-protein-coupled receptor-interacting protein 1), and Scribble^11,14,19,20^. Arhgef7 has been implicated in multiple processes including the regulation of focal adhesion maturation, actin dynamics, the remodeling and trafficking of membranes and exocytosis^11–14,18,19,21–27^.

In the nervous system, Arhgef7 acts as regulator of dendrite branching, the formation of dendritic spines^28–31^ and synaptic structure and function^14,32–35^. Arhgef7 is localized to presynaptic sites and the postsynaptic density by its interaction with Scribble, Git1/2 and Shank (SH3 and multiple ankyrin repeat domains) proteins, respectively^19,35–37^. Arhgef7 regulates actin polymerization by interacting with N-WASP, Pak1 and Rac^29,31,35^. Thereby Arhgef7 promotes the clustering of synaptic vesicles at synapses and maintains the surface levels of GABA_A_ receptors at inhibitory synapses^34,35^. Arhgef7 has also been linked to Ca^2+^-dependent exocytosis and neurotransmitter release^19,25,38,39^.

Much less is known about the function of Arhgef7 in axon formation. The interaction between Paks and Arhgef7 regulates the actin cytoskeleton in growth cones^40,41^. Knockdown experiments in polarized hippocampal neurons revealed a role of Arhgef6 but not Argef7 in axon branching^42^. Here we show that Arhgef7 is essential at early stages of neuronal polarization for axon formation. Arhgef7-deficient neurons are unable to extend an axon in culture and in the developing cortex. The loss of axons can be rescued by the expression of active TC10 but not Cdc42. Our results indicate that Arhgef7 plays an important role in axon formation acting upstream of TC10.

## Results

### Arhgef7 is required for axon formation

To identify GEFs that are required for axon formation we analyzed the function of Arhgef7 during neuronal differentiation. Arhgef7 is expressed in the embryonic brain and its expression increases postnatally^42^ (Fig. 1b). Multiple Arhgef7 isoforms have been described that differ by the presence of the N-terminal CH domain and alternative C-termini^18,43–46^. In the embryonic brain, the largest isoform with a molecular of approximately 100 kDa predominates (Fig. 1a,b), which probably corresponds to an Arhgef7 variant with a CH domain^18,42^. With the increased postnatal expression of Arhgef7 several shorter variants become more prominent with an isoform of approximately 90 kDa being the most strongly expressed one. Staining of cultured neurons from the hippocampus of E18 rat embryos showed that Arhgef7 is present in the soma and at the tip all neurites of unpolarized stage 2 neurons. Upon neuronal polarization it becomes enriched in the axonal growth cone at stage 3 but is present also in the minor neurites (Fig. 1c).

**Figure 2:**
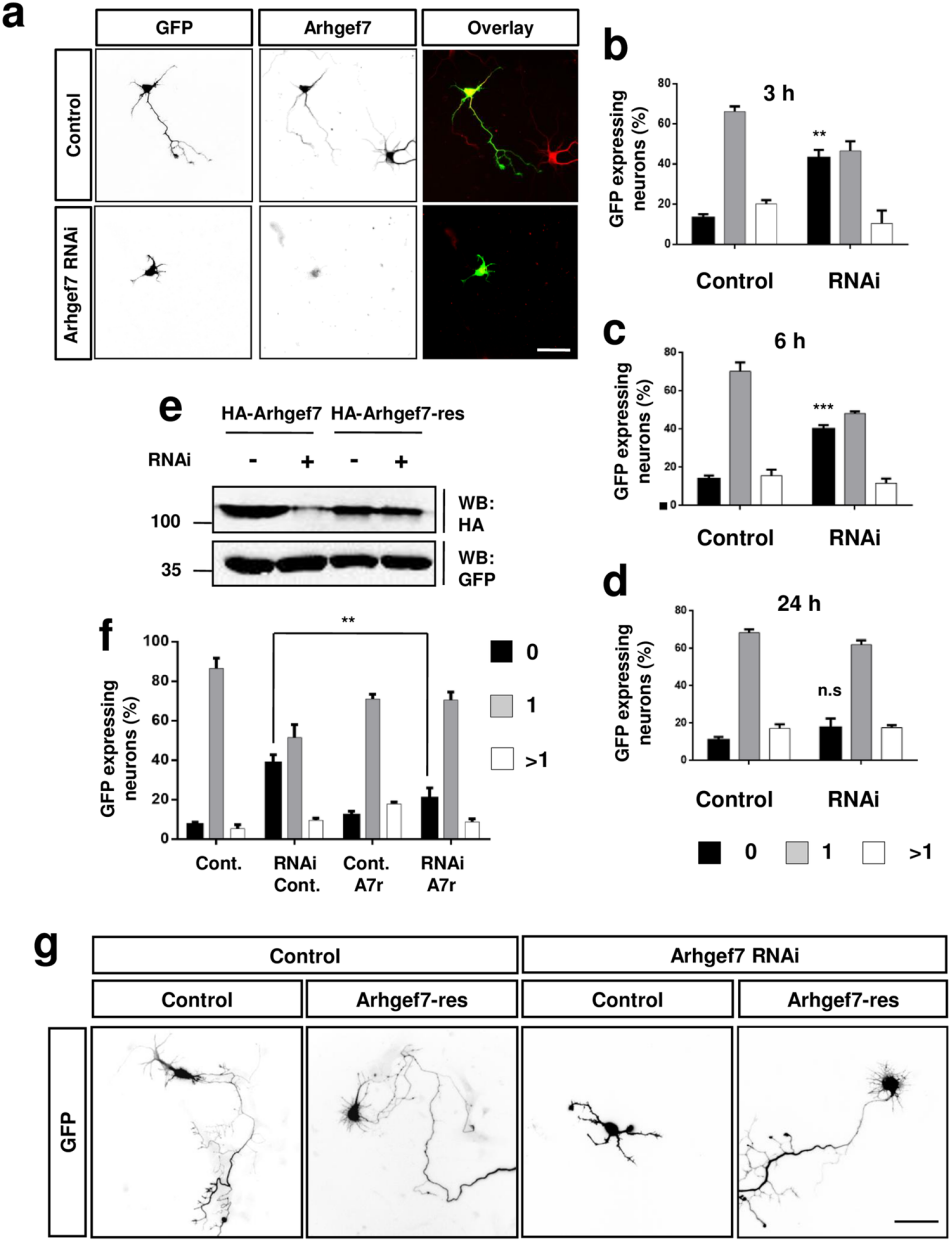
Arhgef7 is required for axon formation. (**a**) Hippocampal neurons were transfected at 6 h with a control vector or vectors for an shRNA against Arhgef7 (Arhgef7 RNAi) and GFP (green) and analyzed at 3 d.i.v. by staining with an anti-Arhgef7 antibody (red). The knockdown results in a loss of Arhgef7 immunoreactivity. (**b**–**d**) Hippocampal neurons were transfected with a control vector or a vector for an shRNA against Arhgef7 (Arhgef7 RNAi) and GFP (green) at 3 (**a**), 6 (**b**) or 24 h (**c**) after platting. The formation of axons was analyzed at 3 d.i.v. by counting the number neurons without an axon (0, black) with a single axon (1, gray), and with multiple axons (>1, white) (Student’s t-Test **p < 0.01, ***p < 0.005 compared to control); at least 50 neurons were counted for each condition (n = 4). (**e**) HEK 293T cells were transfected with a vector for HA-Arhgef7 or RNAi-resistant HA-Arhgef7-res and control vector or a vector for an shRNA directed against Arhgef7, which also express GFP. The expression of Arhgef7 and GFP was analyzed by Western blot (WB) using antibodies against HA and GFP. The molecular weight is indicated in kDa. (**f**,**g**) Hippocampal neurons were transfected with a control vector or a vector for an shRNA against Arhgef7 (RNAi) and GFP (control) or RNAi-resistant HA-Arhgef7-res (A7r) as indicated. The formation of axons was analyzed was analyzed by counting the number neurons without an axon (0, black) with a single axon (1, gray), and with multiple axons (>1, white) (ANOVA, Student’s t-Test, ***p < 0.005, n = 3 with at least 50 neurons counted per condition; **p < 0.01, n = 3). The scale bar is 20 μm.

To investigate if Arhgef7 is involved in axon formation, we performed knockdown experiments with cultured hippocampal neurons using an shRNA that targets all Arhgef7 isoforms. The efficiency of the shRNA construct was verified by Western blot after co-expression with HA-Arhgef7 in HEK 293T cells and by immunofluorescence after transfection of neurons (Fig. 2a,e). Hippocampal neurons were transfected at 3, 6 and 24 h after plating with the shRNA vector and analyzed at 3 d.i.v. by staining with the Tau-1 antibody as axonal and an anti-MAP2 as dendritic marker (Fig. 2a–d). Knockdown of Arhgef7 resulted in an increase in the number of unpolarized neurons from 9 ± 2% in controls to 44 ± 4% after transfection at 3 h (Fig. 2b). Transfection of neurons at 6 h after plating showed a similar result (Fig. 2b,c; control: 14 ± 2% unpolarized neurons, Arhgef7 knockdown: 41 ± 2%). However, no significant effect was observed when neurons were transfected at 24 h after plating (21 ± 4% unpolarized neurons) (Fig. 1d). This indicates that Arhgef7 is required for axon formation and that its function is restricted to early stages of neuronal polarization. This requirement for an early knockdown is consistent with previous studies that did not report defects in axon formation when neurons were transfected after they were already polarized^42^. In order to confirm the specificity of the Arhgef7 knockdown phenotype, we performed rescue experiments with an RNAi-resistant Arhgef7 expression construct (Arhgef7-res) (Fig. 2e–g). The number of unpolarized neurons was increased from 8 ± 3% in controls to 39 ± 4% after expression of the shRNA against Arhgef7 (Fig. 2f,g; n = 3, p < 0.01). After expression of Arhgef7-res together with the shRNA, the number of polarized neurons with a single axon increased from 52 ± 5% after knockdown of Arhgef7 to 71 ± 3% comparable to the value for controls (87 ± 4%, Fig. 2f,g). Expression of Arhgef7 alone did not affect axon formation (neurons with a single axon: 71 ± 2%). These results confirm the specificity of the shRNA directed against Arhgef7. Taken together, our results show that Arhgef7 is required for axon formation.

### Arhgef7 is required for axon formation *in vivo*

**Figure 3:**
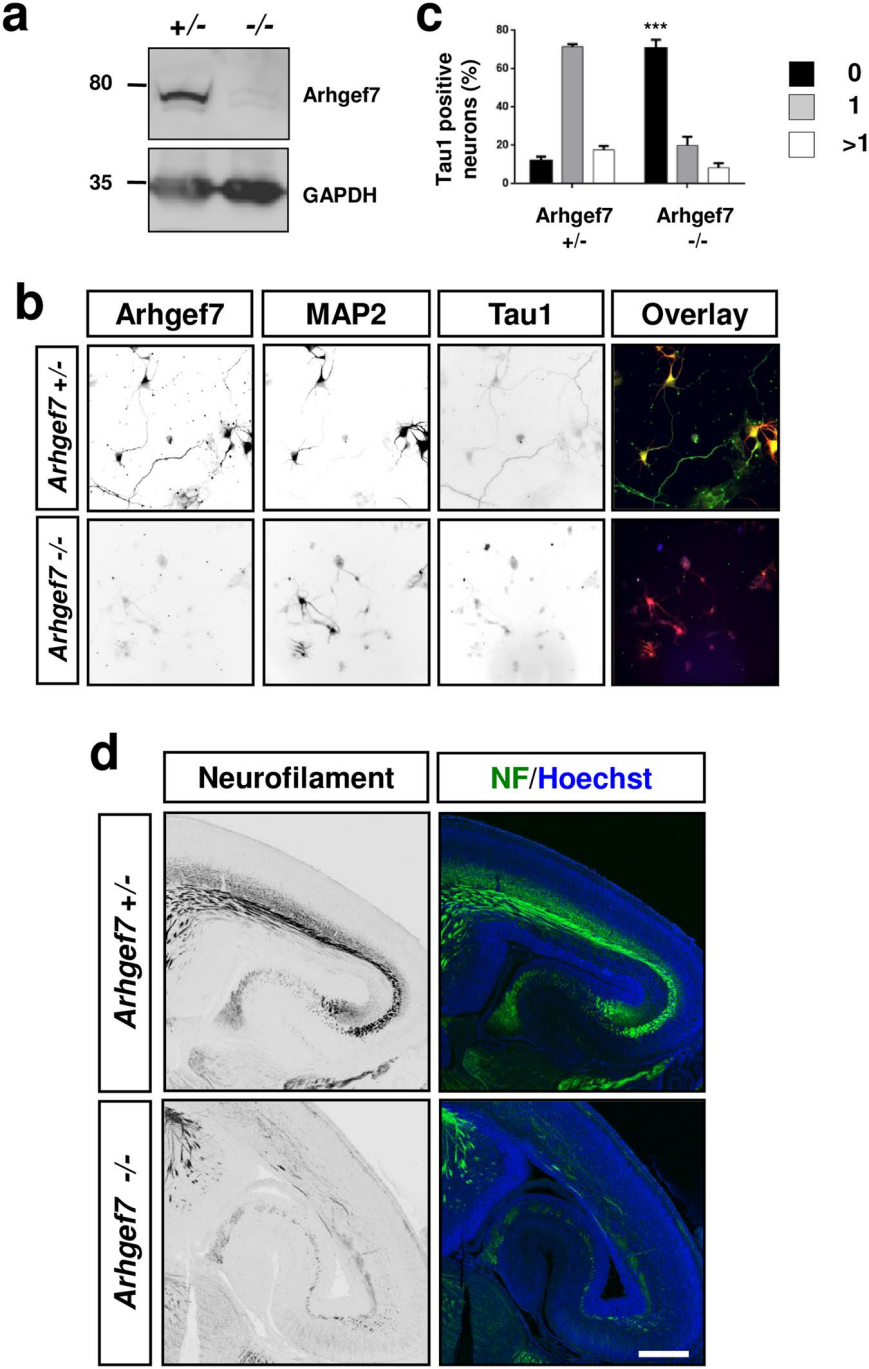
Arhgef7 is required for axon formation in the cortex. (**a**) The expression of Arhgef7 in the cortex from *Arhgef7*^fl/+^; *Emx1*^Cre/+^ (+/−) or *Arhgef*7^fl/fl^; *Emx1*^Cre/+^ (−/−) E17.5 embryos was analyzed by Western blot. Analysis of GAPDH expression confirmed that the loading of comparable amounts of protein. Numbers indicate the molecular weight in kDa. (**b**) Cultured neurons from the cortex of *Arhgef7*^fl/+^; *Emx1*^Cre/+^ (+/−) or *Arhgef*7^fl/fl^; *Emx1*^Cre/+^ (−/−) E17.5 embryos were stained at 3 d.i.v. (stage 3) with an anti-Arhgef7 (green), an anti-MAP2 (red) and the Tau-1 (blue) antibody. (**c**) The formation of axons was analyzed at 3 d.i.v. by counting the number neurons without an axon (0, black) with a single axon (1, gray), and with multiple axons (>1, white). (ANOVA, Student’s t-Test, ***p < 0.005, n = 3 with at least 50 neurons counted per experiment). The scale bar is 20 μm. (**d**) Coronal sections from the brains of E17 *Arhgef*7^fl/fl^; *Emx1*^Cre/+^ (Arhgef7−/−) or *Arhgef7*^fl/+^; *Emx1*^Cre/+^ (Arhgef7+/−) mouse embryos were stained with antibodies specific for the neurofilament medium chain (NF, green) and a nuclear marker (Hoechst 33342, blue). The scale bar is 100 μm.

To address the question if Arhgef7 is required for axon formation also *in vivo* we analyzed the phenotype of a conditional *Arhgef7* knockout. Since a complete knockout of Arhgef7 is embryonically lethal^15^ we generated a cortex-specific knockout using a conditional *Arhgef7* allele (*Arhgef7*^flox^; Suppl. Fig. S1) and the *Emx1-Cre* line, which mediates the deletion in the dorsal telencephalon from E10.5 onwards^47,48^. Western blots confirmed the loss of Arhgef7 in the embryonic cortex of homozygous E17.5 *Arhgef7*^flox/flox^; *Emx1*^Cre/+^ knockout mice (called Arhgef7-cKO hereafter) (Fig. 3a). We cultured cortical neurons from homozygous or heterozygous (*Arhgef7*^flox/+^; *Emx1*^Cre/+^) E17.5 Arhgef7-cKO embryos and analyzed axon formation at 3 d.i.v. by staining with an antibody specific for Arhgef7 and markers for axons (Tau-1) and dendrites (MAP2) (Fig. 3b,c). Cortical neurons from knockout embryos showed an almost complete loss of Arhgef7 expression (Fig. 3b). 75 + 5% of the neurons from homozygous Arhgef7-cKO embryos were unpolarized and did not extend a Tau-1 positive axon compared to 17 ± 3% in cultures from heterozygous embryos (Fig. 3c).

To analyze axon formation in the developing brain, sections from homozygous and heterozygous E17 Arhgef7-cKO embryos were stained with an antibody for neurofilament intermediate chain as axonal marker. The staining revealed a severe loss of axons in the IZ, as well as in the hippocampus (Fig. 3d). The corpus callosum was severely reduced (Suppl. Fig. S2). These results show that Arhgef7 is required for axon formation during cortical and hippocampal development.

### Arhgef7 acts upstream of TC10 during axon specification

**Figure 4:**
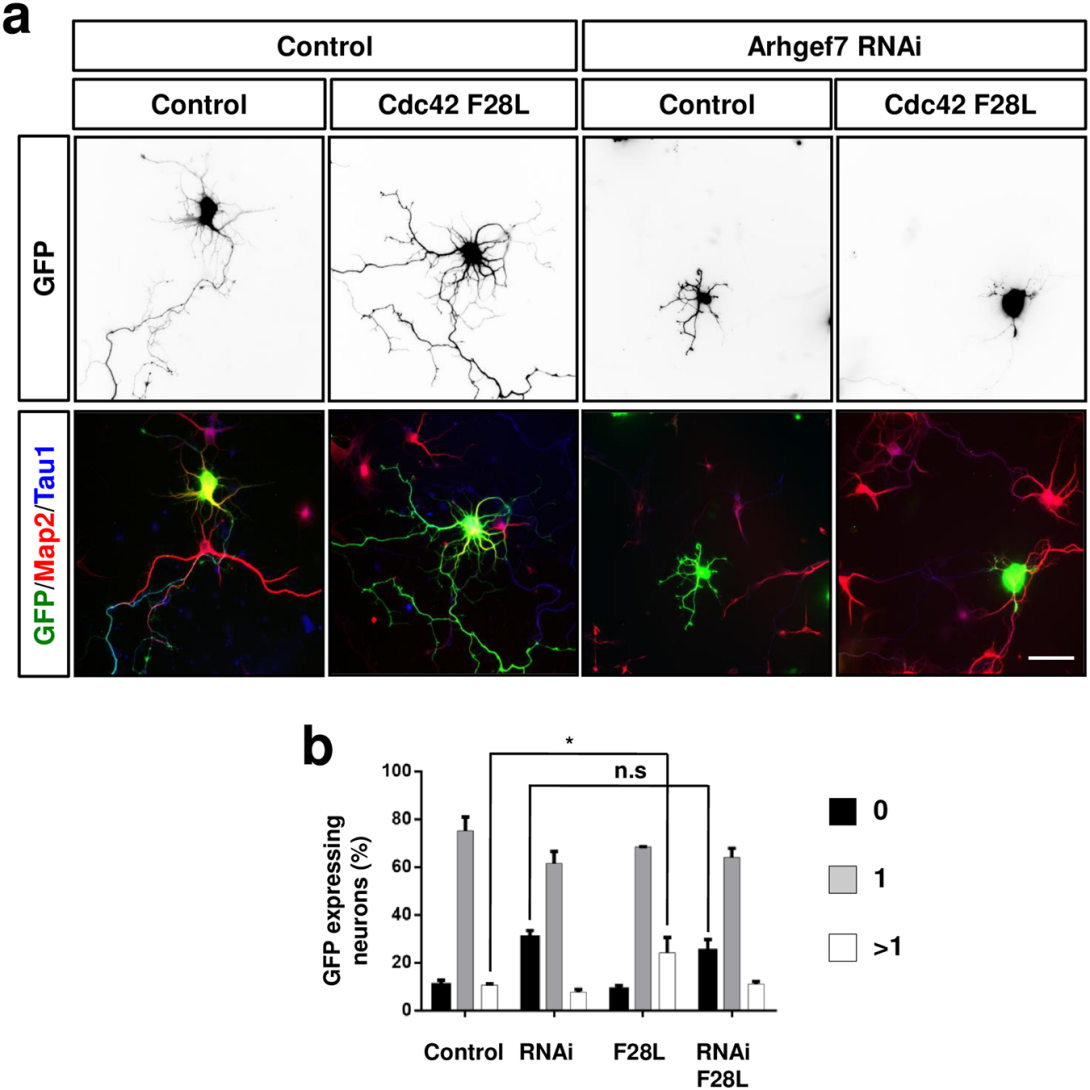
Active Cdc42 is not able to rescue the loss of Arhgef7. (**a**) Dissociated hippocampal neurons were transfected 6 h after plating with expression vectors for an shRNA against Arhgef7 (Arhgef7 RNAi) and GFP-Cdc42F28L or GFP (green). Neurons were analyzed at 3 d.i.v. by staining with an anti-MAP2 (red, dendrites) and the Tau-1 (blue, axons) antibody. The scale bar is 20 μm. (**b**) The formation of axons was analyzed at 3 d.i.v. by counting the number neurons without an axon (0, black) with a single axon (1, gray), and with multiple axons (>1, white) (ANOVA, Student’s t-Test, *p < 0.05, compared to controls, n.s is non significant for the indicated comparison, n = 3 experiments with at least 50 neurons counted per condition).

The role of Arhgef7 in axon development could be mediated by its function as a GEF for Cdc42 that is crucial for axon specification^6,7,9,10^. Therefore, we tested if active Cdc42 is able to rescue the loss of Arhgef7. For these rescue experiments we used a fast cycling Cdc42 mutant (Cdc42 F28L) because constitutively active Cdc42 G12V blocks neurite extension^7^. The expression of Cdc42 F28L at moderate levels slightly increased the number of neurons with multiple axons from 10 ± 1% in controls to 26 ± 5% (Fig. 4a,b, p < 0.05) as previously described^7^. However, the expression of Cdc42 F28L was not able to significantly reduce the number of unpolarized neurons after knockdown of Arhgef7 (control: 10 ± 2%, Arhgef7 shRNA: 38 ± 3%, Arhgef7 shRNA + Cdc42 F28L: 34 ± 4% unpolarized neurons, n = 3, p = 0.64; Fig. 4b). Thus, active Cdc42 is not able to rescue the loss of Arhgef7 during axon formation.

**Figure 5:**
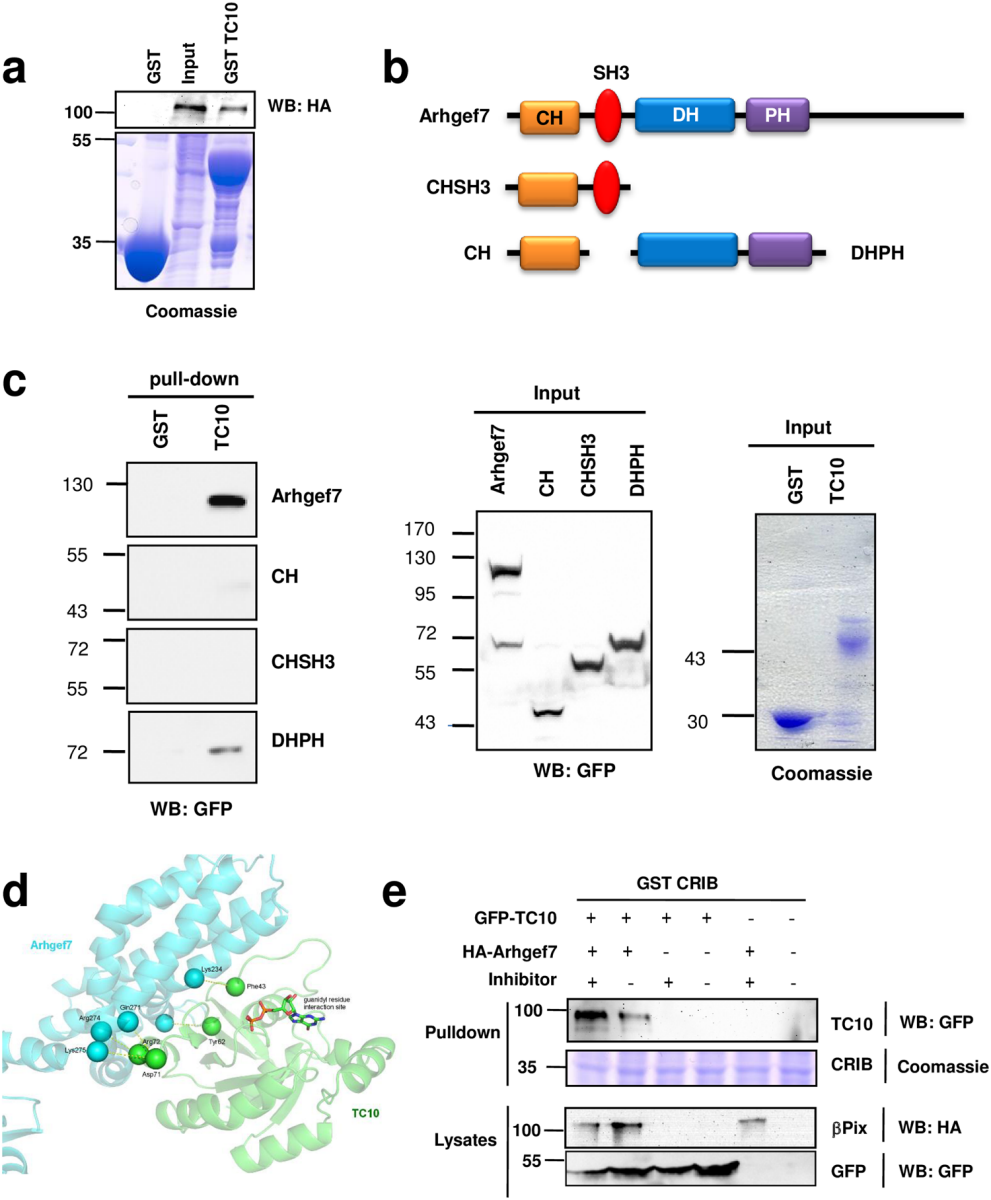
Arhgef7 interacts with TC10. (**a**) Bacterially expressed GST-TC10 was coupled to glutathione-sepharose beads and incubated with lysates of HEK 293T cells transfected with the expression vector for HA-Arhgef7. Bound Arhgef7 was analyzed by Western blot using an anti-HA antibody. The expression of comparable amounts of GST proteins was visualized by Coomassie blue staining. (**b**) Schematic representation of Arhgef7 domains expressed as GFP fusion proteins that were used for pull-down experiments. (**c**) Bacterially expressed GST or GST-TC10 was coupled to glutathione-sepharose beads and incubated with lysates of HEK 293T cells transfected with the expression vectors for GFP-Arhgef7, or GFP-fusion proteins for the CH, CH-SH3 of DH-PH domains as indicated. Bound GFP-fusion proteins and the expression of comparable amounts of protein was analyzed by Western blot using an anti-GFP antibody or Coomassie blue staining. Molecular weights are indicated in kDa. (**d**) Homology models obtained for a complex between Arhgef7 (cyan) and TC10 (green) are shown with Arhgef7 depicted in cyan and TC10 in green. Putative polar interactions between Arhgef7 and TC10 are represented by broken yellow lines connecting alpha-carbon atoms of each residue. (**e**) Bacterially expressed GST or GST-PBD that specifically binds active TC10 was coupled to glutathione-sepharose beads and incubated with lysates of HEK 293T cells transfected with the expression vectors for GFP-TC10 or GFP and HA-Arhgef7 (+) or pcDNA3.1-HA (−) in the presence (+) or absence (−) of a phosphatase inhibitor as indicated. Bound TC10 and the expression of comparable amounts of protein were analyzed by Western blot using anti-HA and anti-GFP antibodies or Coomassie Blue staining. Molecular weights are indicated in kDa.

The GTPase TC10 is a close relative of Cdc42 (82% amino acid sequence similarity), and previous studies have shown that it is an important regulator of axon formation^49,50^. To investigate whether Arhgef7 acts through TC10, we first tested if they interact biochemically. Pull-down assays with bacterially expressed GST-TC10 and HA-Arhgef7 expressed in HEK 293T cells showed that Arhgef7 binds to TC10 (Fig. 5a). To delineate the Arhgef7 domain that interacts with TC10 we performed pull-down assays with GFP-fusion proteins for different Arhgef7 domains expressed in HEK 293T cells (Fig. 5b,c). While full-length Arhgef7 and the DH-PH domain bound TC10 neither the CH nor the combined CH and SH3 domains showed a detectable interaction with TC10 (Fig. 5c). We could not test the C-terminal domain of Arhgef7 because of its poor expression.

The interaction between the DH-PH domain of Arghef7 and TC10 suggests that Arhgef7 acts as a GEF for TC10. Since a structure is not available for Arhgef7 we modeled its DH-PH domain based on that of P-Rex1 (phosphatidylinositol-3,4,5-trisphosphate dependent Rac exchange factor 1) as a template^51,52^. The Rac GEF P-Rex1 is the closest homolog based on amino acid sequence similarity for which a high-resolution structure of a complex between the DH-PH domain and a GTPase is available. All-atom structural models were derived by molecular modeling for the complex of the Arhgef7 DH-PH domain with Rac1, Cdc42 and TC10 (Fig. 5d, Suppl. Fig. S3). These models served to estimate the binding energies for the complexes that were derived. The models yielded a comparable strength of binding between the Arhgef7 DH-PH domain and Cdc42 (estimated binding free energy (ΔG): −67.6 kCal/Mol) or TC10 (−75.2 kCal/Mol) and a slightly lower value for the complex with Rac1 (−44.2 kCal/Mol). These results indicate a high affinity for the interaction between Arhgef7 and TC10. The modeling of the complex between the Arhgef7 DH-PH domain and TC10 suggests the presence of a strong hydrophobic contribution (Suppl. Fig. S4a) coupled with a salt-bridge (Asp71:TC10-Lys275:Arhgef7) and several polar interaction between residues on the interface between the two proteins (Phe43, Tyr62,Arg72 on TC10 and Lys234,Gln271,Arg274 on Arhgef7, Fig. 5e). To investigate the contribution of the potential salt-bridge, we tested the interaction of two TC10 mutants (TC10 D71A, TC10 D71K) with Arhgef7 (Suppl. Fig. S4b). Both mutations reduced the binding modestly but did not abolish it indicating that additional residues make important contributions to the interaction with Arhgef7. Our biochemical characterization together with the free energy values and the 3D reconstruction supports the possibility of an interaction between the DH-PH domain of Arhgef7 and TC10.

In order to test whether Arhgef7 can activate TC10, we transfected HEK 293T cells with expression vectors for TC10 and HA-Arhgef7 and determined the amount of active TC10 using a pull-down assay with the GTPase-binding domain (PBD) from PAK1 (GST-PBD) that binds active TC10^53,54^. Cdc42 was used as a positive control (Suppl. Fig. S5). While little active TC10 was detectable without co-expression of a GEF, a strong signal was apparent upon co-expression with Arhgef7 (Fig. 5e). The addition of a phosphatase inhibitor increased the amount of active TC10 consistent with reports that Arhgef7 activity is regulated by phosphorylation^10,31,55^. These results show that the co-expression of Arhgef7 increased the amount of GTP-bound TC10 indicating that it acts upstream of it.

**Figure 6:**
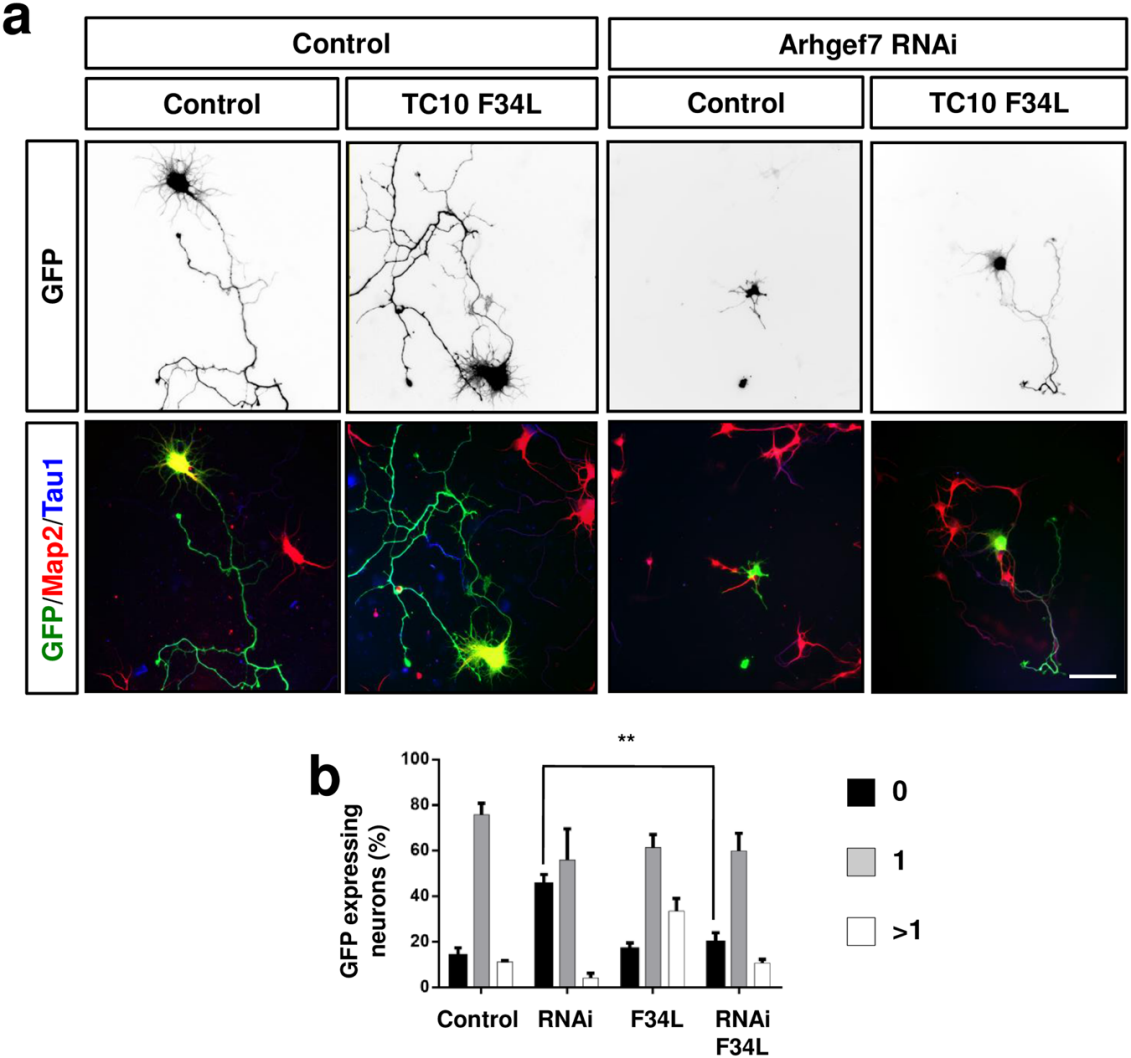
Active TC10 rescues the loss of axons caused in Arhgef7-deficient neurons. (**a**) Dissociated hippocampal neurons were transfected at 6 h after plating with expression vectors for an shRNA against Arhgef7 (Arhgef7 RNAi) and GFP-TC10F34L or GFP (green). Transfected neurons were analyzed at 3 d.i.v. by staining with an anti-MAP2 (red, dendrites) and the Tau-1 (blue, axons) antibody. The scale bar is 20 μm. (**b**) The formation of axons was analyzed at 3 d.i.v. by counting the number neurons without an axon (0, black) with a single axon (1, gray), and with multiple axons (>1, white) (ANOVA, Student’s t-Test, **p < 0.01, n = 3 experiments with 50 neurons counted per condition).

To investigate whether Arhgef7 indeed acts upstream of TC10 to promote axon formation, we tested if active TC10 is able to rescue the loss of Arhgef7. Expression of constitutively active TC10 Q67L completely blocked neurite extension (data not shown) similar to Cdc42 G12V^7^. Therefore, we used the fast cycling mutant TC10 F34L for rescue experiments with hippocampal neurons^56^. Neurons were transfected with vectors for the shRNA against Arhgef7 and TC10 F34L and analyzed axon formation at 3 d.i.v. (Fig. 6a,b). Expression of low levels of TC10 F34L slightly increased the number of neurons with multiple axons (32 ± 6%). Co-expression of TC10 F34L together with the shRNA directed against Arhgef7 rescued the loss of axons and reduced the number of unpolarized neurons from 44.1 ± 3% after knockdown of Arhgef7 to 19 ± 3%, which is comparable to the 14 ± 3% in controls (Fig. 6b). Thus TC10 F34L is able to restore the ability to extend axons in Arhgef7-deficient neurons. These results indicate that TC10 acts downstream of Arhgef7 to promote axon formation.

## Discussion

Here we show that Arhgef7 is essential for the formation of axons. Its inactivation in cultured neurons as well as in the developing cortex results in an extensive loss of axons. This phenotype can be rescued by the expression of active TC10 in cultured neurons but not by active Cdc42 indicating that Arhgef7 acts upstream of TC10 to promote axon formation. Our results identify a new GEF that is essential for axon extension acting through a novel pathway.

Arhgef7 has been shown to regulate dendrite branching, the formation of dendritic spines and synaptic structure and function but no defects in axon formation had been described^28–35,42^. The transfection of neurons at different time points of culture showed that Arhgef7 function is required for axon extension early during neuronal polarization while a knockdown after 24 h of culture does not lead to a loss of axons. This result indicates that the function in axon formation has not been observed before because the loss of Arhgef7 was induced after this critical period in previous studies^42^.

Arhgef7 has been shown to act as a GEF for Cdc42 that is a central regulator of neuronal polarity^6^. A conditional knockout of Cdc42 results in an almost complete loss of axons in the cortex similar to the Arhgef7 knockout^6^. However, active Cdc42 F28L was not able to restore the ability to form axons in Arhgef7-deficient neurons while it can rescue the knockdown of Rap1^7^. By contrast, expression of TC10 F34L rescued the loss of axons after knockdown of Arhgef7. TC10 is closely related to Cdc42 and is required for axon formation^49,53,57^. We show that Arhgef7 interacts with TC10 and increases the amount of GTP-bound TC10 after heterologous expression. Molecular modeling of the complex between the DH-PH domain and TC10 suggests that the binding free energy of this interaction is comparable to that of Cdc42. Taken together, these results indicate that Arhgef7 acts upstream of TC10 to activate it.

Several TC10 effectors are known that could mediate its function in axon formation. These include Par6, Pak1, N-WASP and Exo70^1,53,58–60^. Arhgef7 has been implicated so far mainly in the regulation of the actin cytoskeleton^42,58^. In addition to changes in cytoskeletal dynamics, the expansion of the plasma membrane (PM) by the insertion of specialized vesicles is essential for axon formation^61,62^. The exocytosis of these specialized plasmalemmal precursor vesicles (PPVs) in the growth cone requires the exocyst complex, a conserved octameric complex that mediates the tethering of vesicles at the PM prior to their fusion^49,63^. The exocyst complex is localized to the PM by the Exo70 and Sec3 subunits. Exo70 interacts with active TC10 that recruits it to the PM^50,63,64^. Insulin-like growth factor (Igf1) induces the TC10-dependent recruitment of the exocyst complex and PPVs to the PM to promote axon growth^49,50,61,62,65,66^. The knockdown of TC10 or Exo70 impairs the polarized insertion of PPVs in the growth cone and prevents formation of axons in cultured hippocampal neurons^49^.

TC10 also interacts with Par6 that forms a complex with Par3 and aPKC^1,60,67^. Interestingly, the Par complex has been linked to exocyst function by the interaction of Par6 with the exocyst subunit Exo84^68^ and Par3 with Exo70^69^. A function in regulating membrane expansion through the exocyst complex would be consistent with previous studies that implicate Arhgef7 in the regulation membrane trafficking and exocytosis in different cell types^18,19,21,22,25,26,38,55,70^. Taken together our results identify a novel signaling pathway that promotes axon formation through Arhgef7 and TC10. Arhgef7 may act not only by regulating actin dynamics but also membrane expansion through TC10 and the exocyst complex. Future studies will identify the precise molecular mechanism that determines which downstream target mediates the multiple functions of Arhgef7.

## Materials and Methods

### Antibodies

For Western blots, the following antibodies were used: rabbit anti-Arhgef7 (Cell Signaling, #4515, 1:500), rabbit anti-Arhgef7 (Millipore, #07-1450, 1:500), mouse anti-GFP (antibodies inc. 75–131, 1:1000), rabbit anti-GAPDH (Sigma, #G9545, 1:1000), anti-HA (Roche, #1867423, 1:500). For immunofluorescence, we used anti-Arhgef7 (Millipore, #07-1450, 1:200), rabbit anti-NF medium chain (Abcam #ab64300, 1:200), mouse Tau-1 (Chemicon #MAB3420; 1:500), mouse anti-MAP2 (Chemicon #AB5622; 1:1000), Tuj 1 (R&D Systems #MAB1195, 1:1000), and goat secondary antibodies labeled with Alexa 488 or 594 (Molecular Probes). Nuclei were stained with Hoechst 33342 (Molecular probes #C2110, 1:6000). CellTracker Blue CMAC (Molecular Probes, 1:1000 of 10 mM solution) was used as a cell volume marker.

### Plasmids

The Arhgef7 expression vectors were generated from mKIAA0142 (corresponding to NM_001113517.1) by amplifying the coding sequence by PCR and cloning it into the pEGFP-C1 (Clontech) or pcDNA3.1-HA (Invitrogen) vectors. The sequences encoding the different Arhgef7 domains were amplified by PCR and cloned into the pEGFP-C2 vector. The vectors for Cdc42 F28L and pGST-PBD have been described previously^7,8,54^. The coding sequence for mouse TC10 was amplified by PCR and cloned into pGEX4-T2 and pEGFP-N1. The fast cycling mutant TC10 F34L^7,56^ was generated by site directed mutagenesis using the QuikChange Site-Directed Mutagenesis kit (Stratagene) with the primers 5′-AACGACGCCT TACCCGAGG AGTACG-3′ and 5′-TCCTCGGGTA AGGCGTCGTT GGC-3′. To generate an shRNA vector directed against Arhgef7 (Arhgef7 RNAi) an shRNA with the target sequence 5′-AGGGAGTGAG GGAGAGAACG-3′ was cloned into the Xho I and Eco RI sites of the pCAGGS-U6 vector^71^. An RNAi-resistant Arhgef7 vector was created by introducing 2 synonymous mutations into the binding site for the shRNA in Arhgef7 (Arhgef7-res) by site directed mutagenesis using the QuikChange Site-Directed Mutagenesis kit (Stratagene) according to the manufacturer’s instructions with the primers 5′-GGTTTCATCT ATCAGGGAAA GCTGCCGACA ACGGGAATG-3′ and 5′-TGTGATTGTC ATTCCCGTTG TCGGCAGCTT TCCCTGATA-3′.

### Culture of primary cortical and hippocampal neurons

Dissociated cortical and hippocampal neurons from embryonic day 18 (E18) rat or E17 mouse embryos were prepared and transfected by calcium phosphate co-precipitation as described previously^5,71^. Neurons were plated at 70,000 cells per well in a 24-well plate coated with poly-L ornithine in Neurobasal medium (Life Technologies) and cultured at 37 °C and 5% CO_2_. The culture medium was replaced by 400 μl Opti-MEM (Life Technologies) before the DNA/ CaCl_2_ mixture was added. After incubation for 45 min at 37 °C and 5% CO_2_ the neurons were washed for 15 min with 1 ml opti-MEM, which had been pre-incubated at 37 °C and 10% CO_2_, and Neurobasal medium was added back to the cells.

### Arhgef7 conditional knockout mouse

After screening a mouse genomic library (129/Sv, RZPD Center, Berlin) with an *Arhgef 7* cDNA, a targeting vector for a conditional knockout was constructed by flanking exon 4 with loxP sites (Suppl. Fig. S1). R1 (129/Sv) ES cells were electroporated with the targeting construct and a knockout line was generated by blastocyst injection of validated ES cells. After crossing to a C57/B6L FLPo-deleter mouse line^72^ to remove the selection cassette mice were backcrossed to the C57BL/6 J background for more then 10 generations. *Emx1*-*Cre* mice^47^ were obtained from The Jackson Laboratory (Bar Harbor, Maine) and crossed with *Arhgef7*^flox/+^ mice. *Arhgef7*^flox/+^; *Emx1*^Cre/Cre^ mice were crossed with *Arhgef7*^flox/flox^ animals to obtain heterozygous (*Arhgef7*^flox/+^; *Emx1*^Cre/+^) and homozygous knockout mice (*Arhgef7*^flox/flox^; *Emx1*^Cre/+^). All mouse strains were maintained in a C57Bl/6 background. Genotyping was done by PCR using the following primers.: 5′-AGGGAGTGAG GGAGAGAACG-3′ and 5′-GTCAGACTGCAACCCAGGAG-3′ for *Arhgef7* and 5′-AAGGTGTGGT TCCAGAATCG-3′, 5′-CTCTCCACCA GAAGGCTGAG-3′, 5′-GCGGTCTGGC AGTAAAAACT ATC-3′ and 5′-GTGAAACAGC ATTGCTGTCA CTT-3′ for *Emx1*. Mice were housed at four to five per cage with a 12-h light/dark cycle (lights on from 07:00 to 19:00 h) at constant temperature (23 °C) with *ad libitum* access to food and water. All animal protocols were carried out in accordance with the relevant guidelines and regulations and approved by the Landesamt für Natur, Umwelt und Verbraucherschutz Nordrhein-Westfalen.

### Immunofluorescence staining of neuronal cultures

Primary cultures of dissociated hippocampal neurons were fixed at 3 days *in vitro* (d.i.v.) with 4% paraformaldehyde/15% sucrose in phosphate buffered saline (PBS) for 20 min, permeabilized with 0.01% Triton X-100/0.1% Na-Citrate/PBS for 10 min on ice and stained with primary and secondary antibodies in 10% NGS/PBS. A Zeiss LSM 700 or LSM 800 confocal laser scanning microscope was used for imaging. Image analysis was done using ImageJ 1.45 s (NIH), and Adobe Photoshop CS5. The stage of neuronal differentiation was determined as described previously^5^.

### Biochemistry

The transfection of HEK 293T cells using the calcium phosphate co-precipitation method, pull-down assays, immunoprecipitation and Western blots were performed as described previously^73^. Transfected HEK 293T cells were lysed in TLB lysis buffer (Tris/HCl 50 mM, pH 7,4, NaCl 150 mM, DTT 1 mM, MgCl_2_ 1,5 mM, EDTA 4 mM, Glycerol 10% (v/v), Triton X-100 1% (v/v), cOmplete protease inhibitor (Sigma-Aldrich) at 4 °C for 30 min. The cell lysate was incubated with antibody at 4 °C for 4 h or overnight and bound proteins precipitated with protein G agarose beads (ThermoFischer Scientific). Bound proteins were eluted with 2x SDS sample buffer and analyzed by Western blot. To determine the amount of active TC10 pull-down assay were performed with the GTPase-binding domain (PBD) from PAK3 (GST-PBD)^8,53,54^. GST fusion proteins were expressed in *E*. *coli* BL21 cells and coupled to glutathione sepharose beads (GE Healthcare). The beads were incubated with lysates of transfected HEK 293T293T cells the bound proteins were eluted with 2x SDS sample buffer and analyzed by Western blot.

For the detection of endogenous proteins, cortices were dissected from mouse embryos at E17. The brains were homogenized in ice-cold modified RIPA buffer (1% IGEPAL, 1% sodium deoxycholate, 0.1% SDS, 50 mM HEPES (pH 7.4), 150 mM NaCl, 10% glycerol, 1.5 mM MgCl_2_, cOmplete protease inhibitor (Sigma-Aldrich) using a glass homogenizer. After incubation for 30 min at 4 °C, the insoluble material was pelleted by centrifugation at 13,000 rpm for 30 min at 4 °C. Western blot analysis was performed using horseradish peroxidase conjugated secondary antibodies and the enhanced chemiluminescence detection system (Uptima, Interchim UP99619A) using the Image Reader LAS-1000 system (Fujifilm).

### Molecular Modeling

Protein structures were downloaded from the Protein Data Bank (PDB, www.rcsb.org). Homology modeling was done using Modeller v 9.18^74^, setting MD refinement to “refine.slow” and leaving the remaining parameters at default. Sequence alignment was performed using Clustal Omega^75^. Only the PH-DH domain of the GEFs were modeled due to the absence of high quality structures for the remaining protein domains, the function of the PH-DH domains as the catalytic domain of GEFs and to simplify the model in terms of calculation complexity. Given the high conservation of amino acid sequences between the human and murine orthologs and the scarcity of available high-resolution structures of murine GTPase/GEF complexes, the structure for the human protein (PDB: 4YON) was used as template. The structures were modeled for the human amino acid sequences. Potential energy minimization was performed on each GTPase/GEF complex structure with GROMACS 4.6 through a multi-step conjugate gradient algorithm using Amber99^76^ as force field. A first minimization was performed fixing all atoms of the GEFs counterpart but those at a close distance (4.5 A) from the GTPase subunit, which was not fixed. The minimization procedure automatically stopped when the resulting structure reached an RMSD threshold of 0.05. A second minimization was performed unfixing all atoms, using the same force field and RMSD threshold. Estimates of the Free Energy of binding of each complex was measured using autodockVina^77^.

### Statistical analysis

Statistical analyses were done using the GraphPad Prism 6.0 software. Statistical significance was calculated for at least three independent experiments using one-way ANOVA, and Student’s t-Test for parametric and Kruskall-Wallis for non-parametric data sets. Significance was defined as: p > 0.05, ns; **p < 0.01, ***p < 0.001.

### Data availability statement

The datasets analyzed during the current study are available from the corresponding author on reasonable request.

## Electronic supplementary material

Supplementary Figures S1 - S13
