# Supplementary material for: The guanine nucleotide exchange factor Arhgef7/βPix promotes axon formation upstream of TC10
Source: Sci Rep. 2018 Jun 11;8:8811. doi: 10.1038/s41598-018-27081-1 (PMC5995858; doi:10.1038/s41598-018-27081-1)
Supplement: Supplementary file 1 — Supplementary Figures S1 - S13 [file 41598_2018_27081_MOESM1_ESM.pdf]

The guanine nucleotide exchange factor Arhgef7/βPix promotes axon formation upstream of TC10

Supplementary Material

Alejandro López Tobón[1,2,3,4], Megalakshmi Suresh[1,2], Jing Jin[1,2], Alessandro Vitriolo[3,4], Thorben Pietralla[1], Kerry Tedford[5], Michael Bossenz[5], Kristina Mahnken[1], Friedemann Kiefer[2,6,7], Giuseppe Testa[3,4], Klaus-Dieter Fischer[5], Andreas W. Püschel[1,2]

[1]Institut für Molekulare Zellbiologie, Westfälische Wilhelms-Universität, Schloßplatz 5, D-48149 Münster, Germany

[2]Cells-in-Motion Cluster of Excellence, University of Münster, D-48149 Münster, Germany
[3]Department of Oncology and Hemato-Oncology, University of Milan, Milan, 20122, Italy
[4]European Institute of Oncology, Via Adamello 16, 20139 Milan, Italy
[5]Institut für Biochemie und Zellbiologie, Otto-von-Guericke-University, Medical Faculty, Leipziger Str. 44, 39120 Magdeburg 39120, Germany
[6]Max-Planck-Institute for Molecular Biomedicine, Mammalian cell signaling laboratory, Röntgenstr. 20, D-48149 Münster, Germany
[7]European Institute for Molecular Imaging, Westfälische Wilhelms-Universität, Waldeyerstr. 15, D-48149 Münster, Germany

Correspondence: apuschel@uni-muenster.de

Supplementary Figures

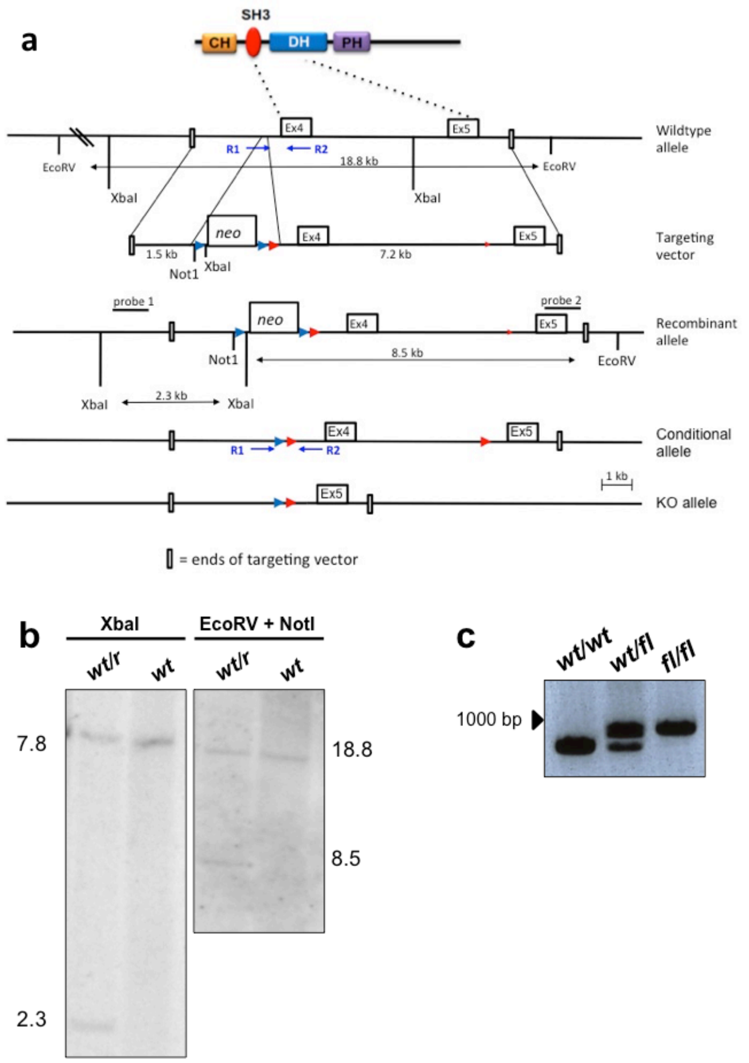

Figure S1. Generation of *Arhgef7* deficient mice. (**a**) A schematic representation of the Arhgef7 domain structure and the *Arhgef7* genomic locus is shown. The targeting vector was constructed to achieve a conditional knockout by flanking exon 4 with loxP sites. The targeted recombinant allele, the conditional (*Arhgef7^{flox}*) and the deleted knockout (KO) *Arhgef7* allele are shown. Cre-mediated recombination removes exon 4 encoding parts of the SH3 domain. Deletion of exon 4 results in a frameshift with a Stop codon after 11 amino acids affecting all isoforms. Exons are represented by open boxes and the FRT and loxP recombination sites by blue and red arrowheads, respectively. The position of the *EcoRV, NotI*, and *XbaI* restriction sites with the predicted sizes of fragments generated by digestion with *XbaI* or *EcoRV* and *NotI*, the short and long arm probes for Southern blot analysis and the R1/R2 primers for genotyping by PCR are indicated. (**b**) Wild type (wt) ES cells and a recombinant ES cell clone (wt/r) used to establish the knockout line were analyzed by Southern blot. After digestion of genomic DNA with EcoRV and NotI, an 18.8 kb fragment for the wild type allele and a 7.2 kb fragment for the recombinant allele are detected by probe 2. A 7.8 kb fragment for the wild type allele (fragment not indicated) and a 2.3 kb fragment for the recombinant allele are detected by probe 1 after digestion with XbaI. (**c**) PCR-based genotyping of the wild type (845 bp fragment) and conditional alleles (971 bp fragment) was done for wild type, heterozygous and homozygous mice using the R1/R2 primer pairs indicated in a.

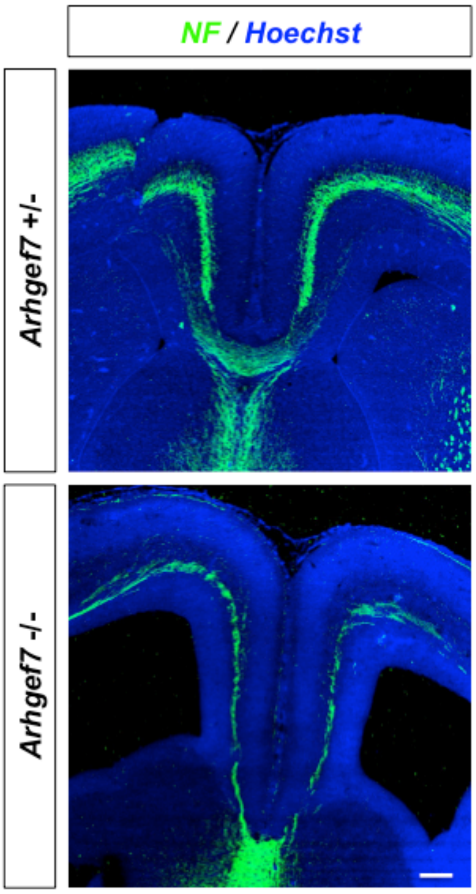

Figure S2. The corpus callosum is severely reduced in the *Arhgef7* mutant. Coronal sections from the brains of E17 *Arhgef7*$^{fl/fl}$;*Emx1*$^{Cre/+}$ (Arhgef7-/-) or *Arhgef7*$^{fl/+}$;*Emx1*$^{Cre/+}$ (Arhgef7+/-) mouse embryos were stained with an antibody specific for neurofilament medium chain (NF, green) and a nuclear marker (Hoechst 33342, blue). The scale bar is 100 μm.

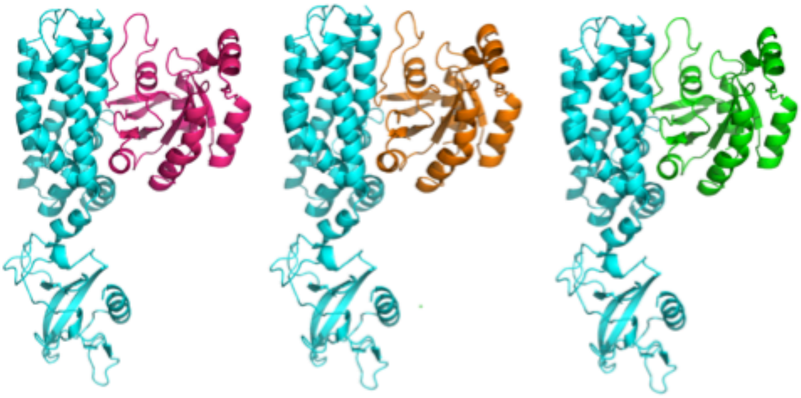

Figure S3. Homology models for Arhgef7/GTPase complexes. Homology models for complexes between Arhgef7 (cayn) and Cdc42 (purple), TC10 (green) and Rac1 (orange) are shown.

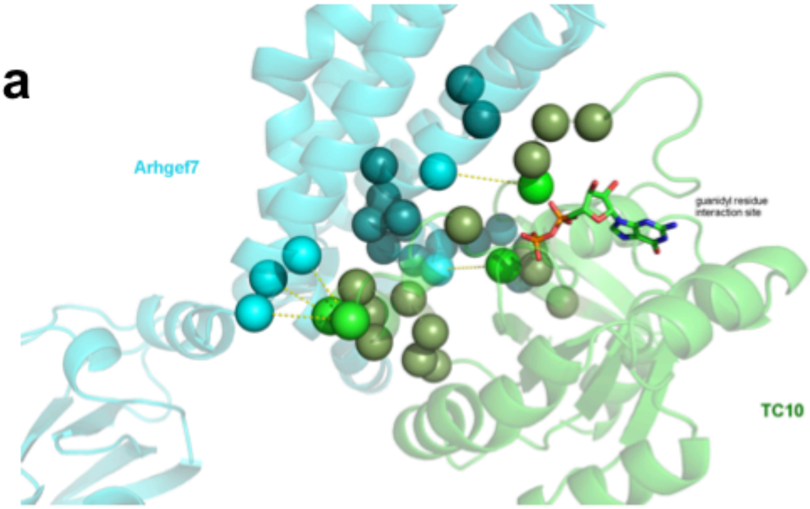

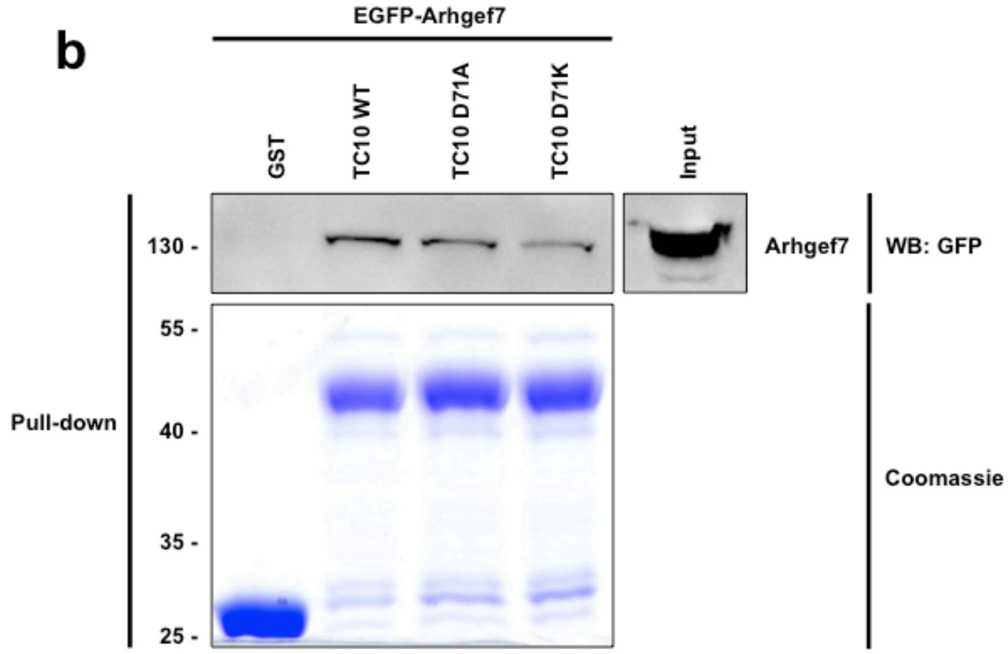

Figure S4. Hydrophobic patches at the interface between Arhgef7 and TC10. (**a**) Hydrophobic patches at the interface between Arhgef7 and TC10 derived from the homology model are indicated. Alpha-carbon atoms of hydrophobic residues are depicted as spheres. Arhgef7 residues are dark cyan and TC10 residues are dark green. (**b**) Bacterially expressed GST, GST-TC10, -TC10-D71A or -TC10-D71K was coupled to glutathione-sepharose beads and incubated with lysates of HEK 293T cells transfected with the expression vector for HA-Arhgef7. Bound Arhgef7 was analyzed by Western blot using an anti-HA antibody. The expression of comparable amounts of GST proteins was visualized by Coomassie blue staining. Molecular weights are indicated in kDa.

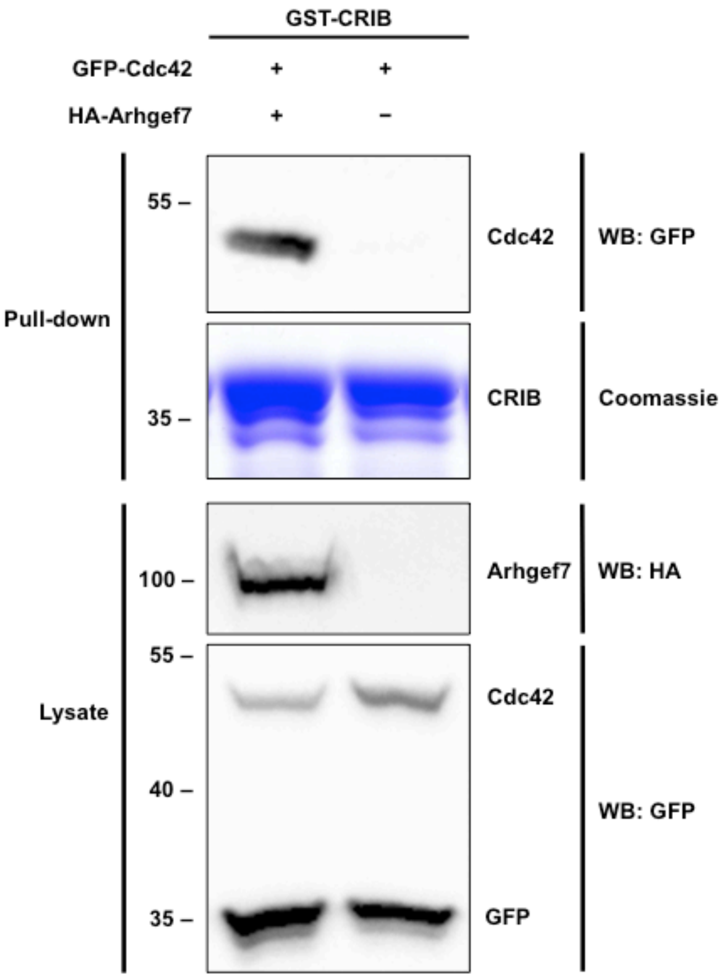

Figure S5. Arhgef7 activates Cdc42. Bacterially expressed GST or GST-PBD that specifically binds active Cdc42 was coupled to glutathione-sepharose beads and incubated with lysates of HEK 293T cells transfected with the expression vectors for GFP, GFP-Cdc42 and HA-Arhgef7 (+) or pcDNA3.1-HA (-, control) as indicated. Bound Cdc42 and the expression of comparable amounts of protein were analyzed by Western blot using anti-HA and anti-GFP antibodies or Coomassie Blue staining. Molecular weights are indicated in kDa.

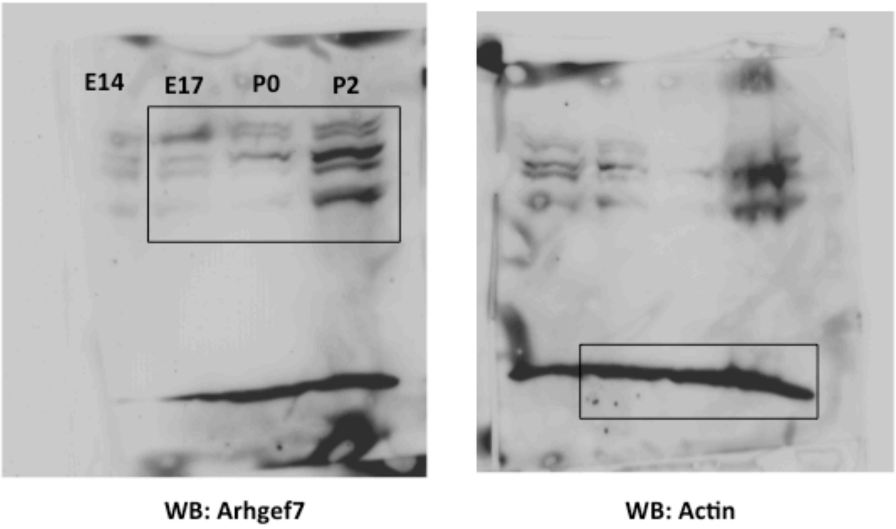

Figure S6. Full-length blots for Figure 1b.

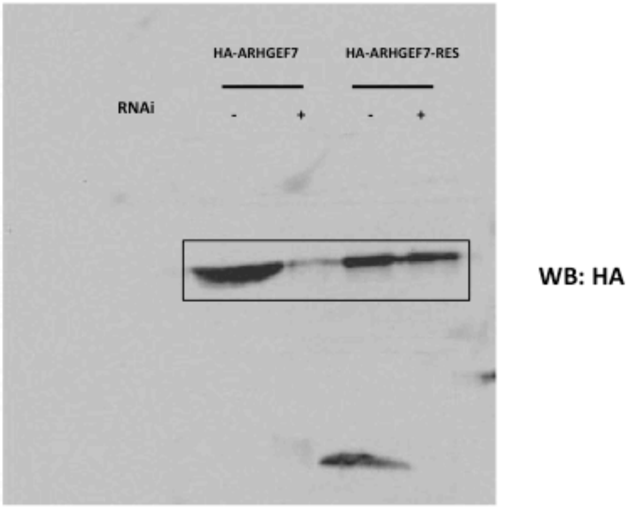

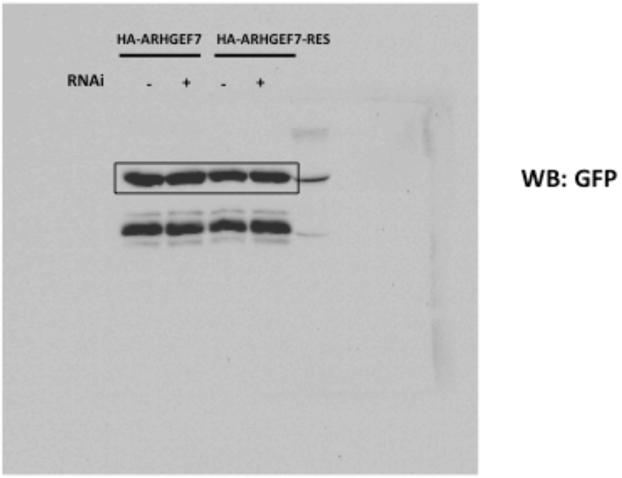

Figure S7. Full-length blots for Figure 2e.

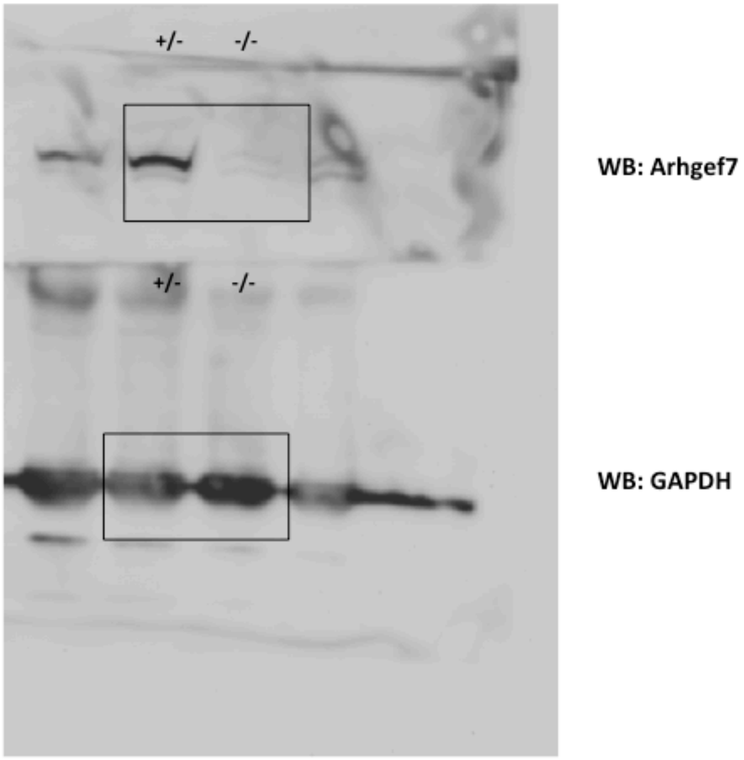

Figure S8. Full-length blots for Figure 3a.

Fig. 5a

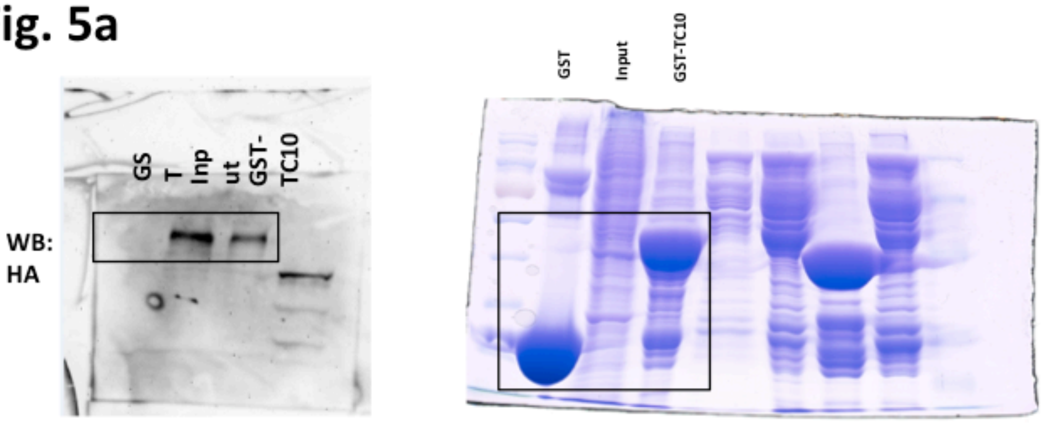

Fig. 5c

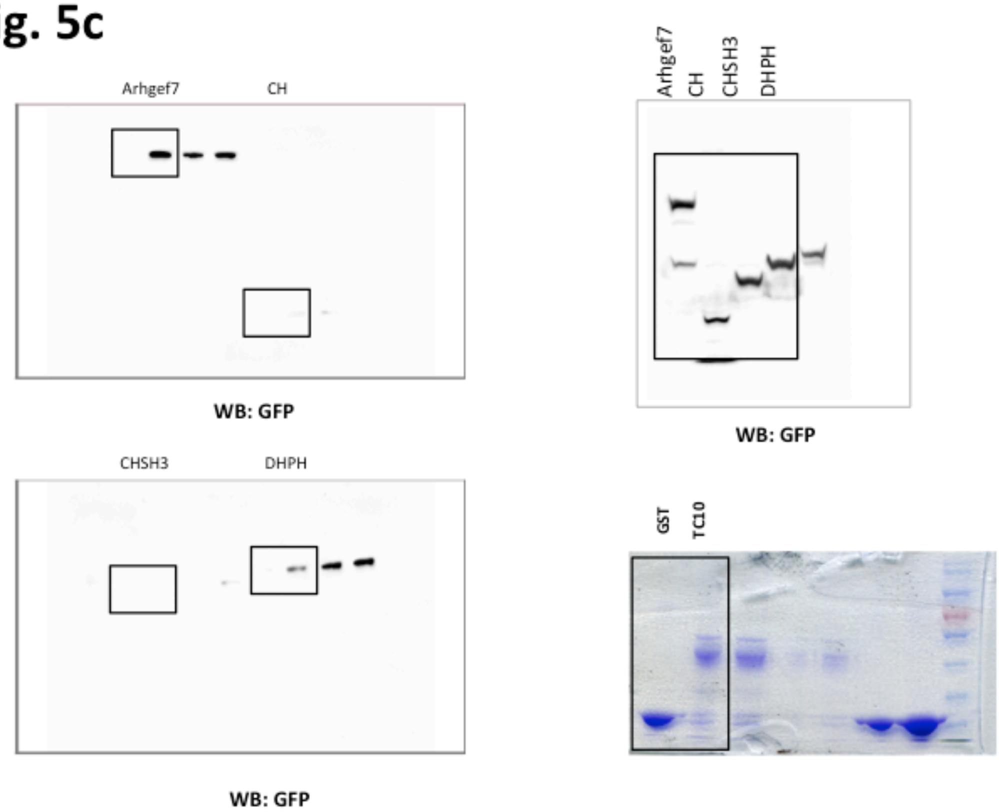

Figure S9. Full-length gels and blots for Figure 5a, c.

Fig. 5e

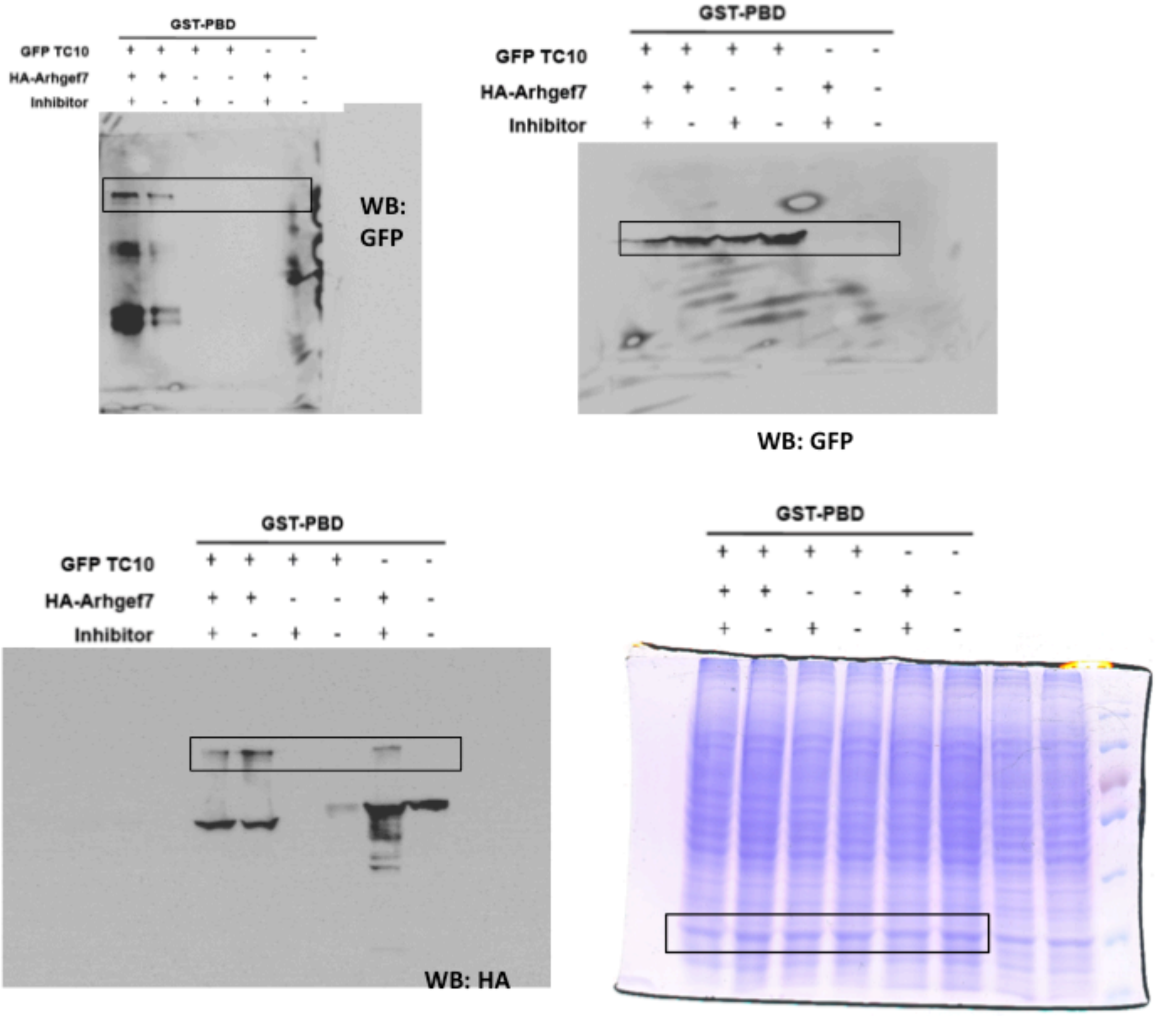

Figure S10. Full-length gel and blots for Figure 5e.

a

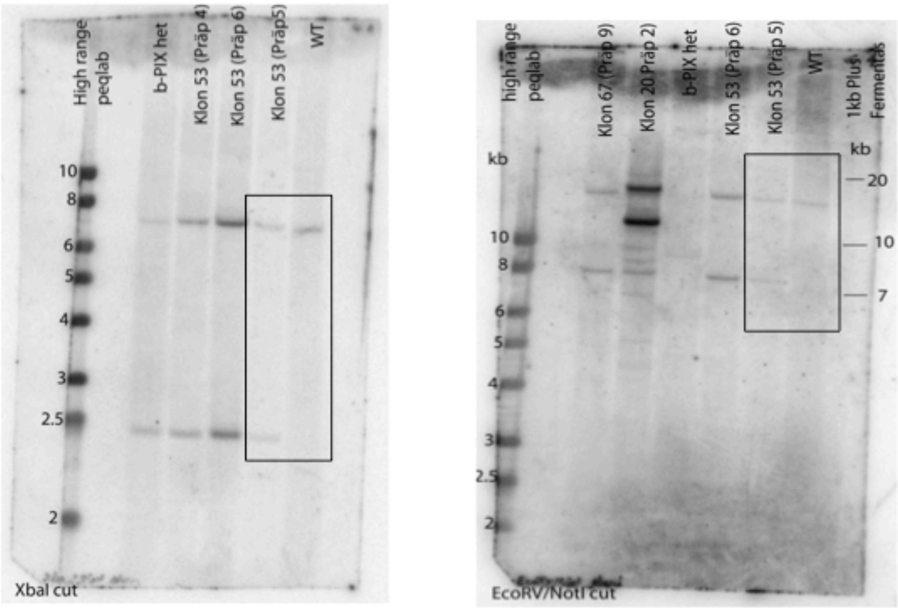

b

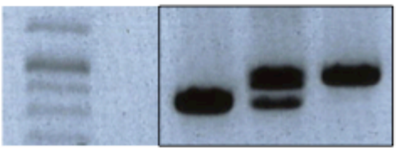

Figure S11. Full-length gels and blots for Figure S1.

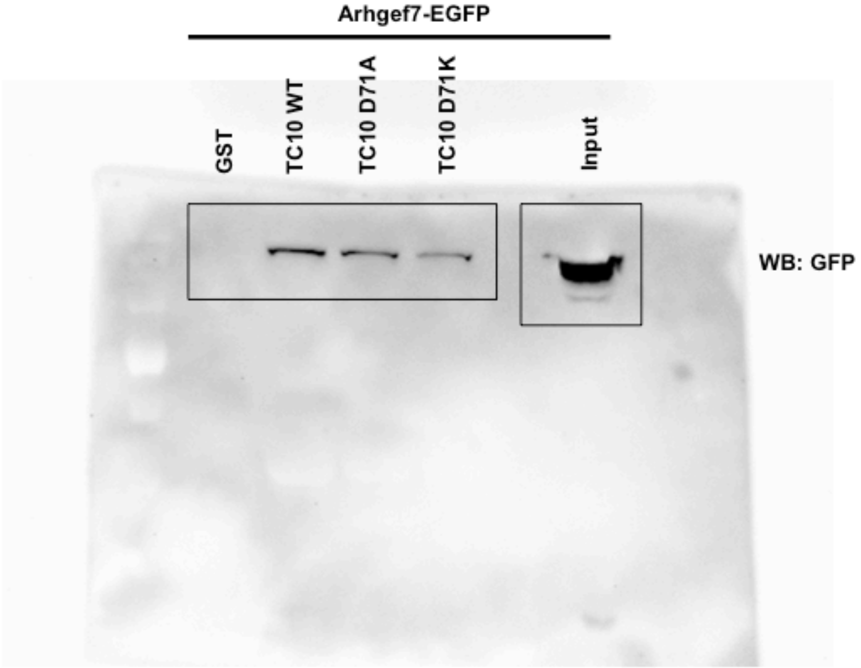

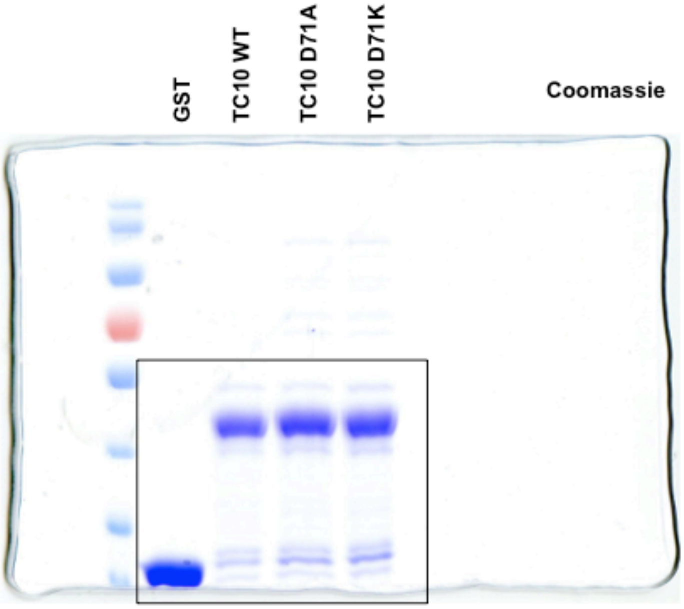

Figure S12 Full-length gels and blots for Figure S4.

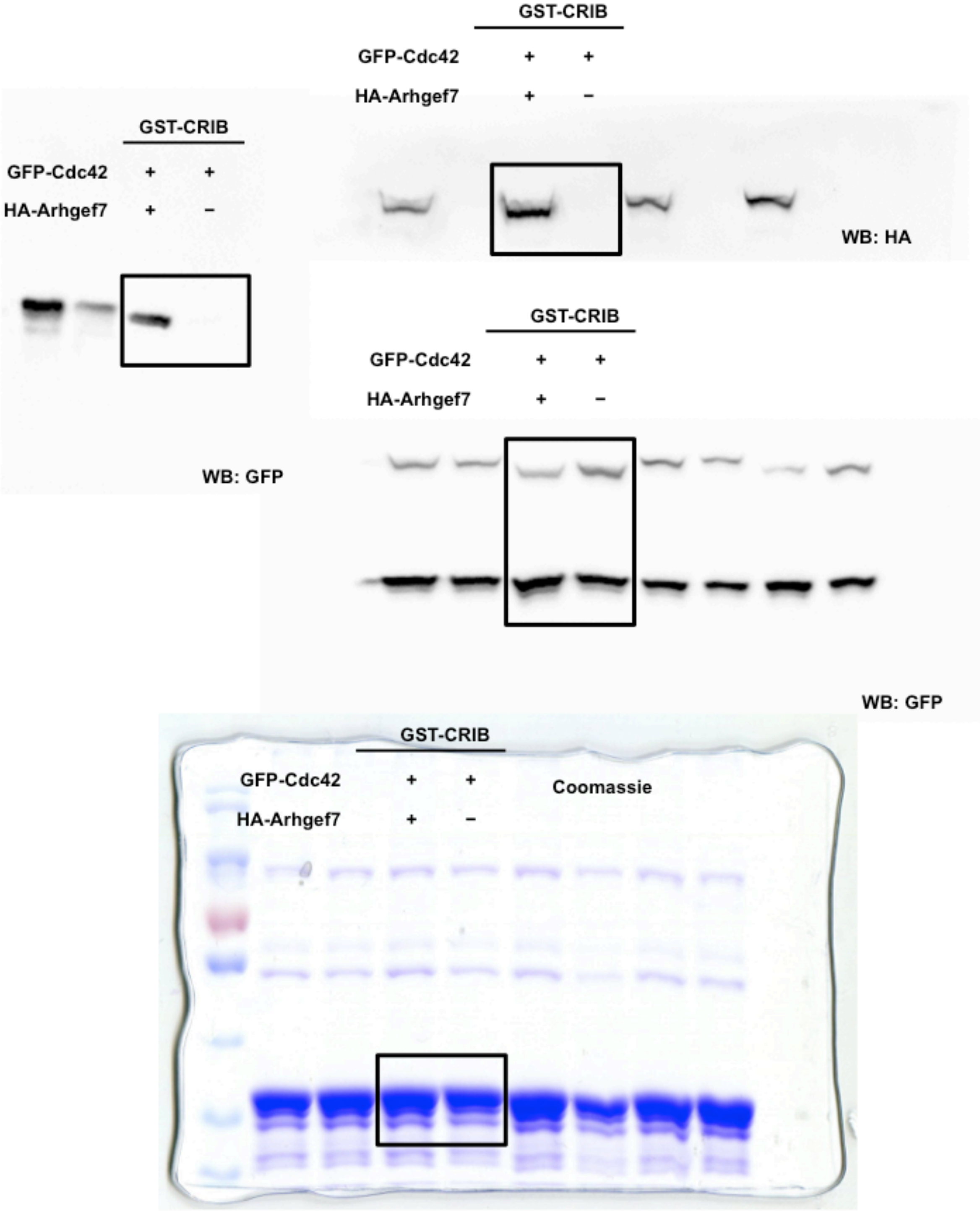

Figure S13. Full-length gels and blots for Figure S5.
